# Entitlement in medical education: an ongoing discourse

**Published:** 2016-10-18

**Authors:** Sylvia R. Cruess, Richard L. Cruess, Yvonne Steinert

**Affiliations:** 1 The Centre for medical education of McGill University

How does medicine both identify and preserve its traditional values in a world in which the practice of medicine, societal values, and the aspirations of each new generation entering the medical profession are changing? Dr. Lester Liao, in his letter to the Editor of the Canadian Medical Education Journal, raises several issues relevant to this question, and he has initiated a discourse that is important to the future practice of medicine. Dr. Liao very eloquently documents his concerns about the appropriateness of some attitudinal changes expressed by his own generation that appear to be acceptable to, and even endorsed by, medicine’s educational establishment. He specifically refers to his generation’s sense of entitlement (a mindset that feeds their narcissism), a belief that they should be able to exert control over the educational process, and the dominant impact of lifestyle on student decision-making. Clearly these attitudes are inconsistent with his concept of the “good doctor”. He also describes how this sense of entitlement is often nurtured or reinforced by faculty leadership and clinical role models.

To begin, we would like to point out that medical education has been in flux throughout its history, with change being the hallmark since the emergence of the modern medical profession in the middle of the 19^th^ century.^1^,^2^ Indeed, it has been pointed out that without a certain amount of intergenerational disagreement, progress would be impossible.^3^ While the current discussion over work hours at both the undergraduate and postgraduate levels are relevant and important, the issue itself stretches back through the ages. The Halstead surgical training program in the pre-World War II era at Johns Hopkins University required residents to be on call in the hospital for the five years of the training program, and often beyond. If they expressed dissatisfaction, they “lacked commitment.”^2^ This clearly required redress and, just as obviously, constituted an intergenerational disagreement; its modification represented progress. One must also record the positive impact of a change in the status of students and residents in the medical hierarchy. While a hierarchical structure does, and indeed must, continue to exist, the emphasis on learning and the learning environment,^4^ as well as the role of the learner as a future member of medicine’s community of practice,^5^ has clearly aimed to improve both the quality of medical education and the student experience.

As we have implied, this issue is not new, having been present for many decades. A 1986 editorial in the New England Journal of Medicine entitled “Coping with entitlement in medical education” stated that, in spite of changes in the content of the medical curriculum over time, problems with entitlement still existed. The author suggested “placing emphasis on the more selfless aspects of the physician’s identity”^6^ - an approach that still resonates today.

Dr. Liao clearly believes that current practices have resulted in unintended consequences that are detrimental to medicine. He actually is continuing a discourse that is, in part, being categorized as an intergenerational disagreement.^7^ Refreshingly, he does not ascribe all of the blame to older generations, saving his more direct criticism for his own generation. Moreover, although he emphasizes the very positive role of some role models and mentors, he does accuse medicine’s educational establishment of fostering and indeed enabling attitudes and behavior patterns characteristic of his generation.

We applaud Dr. Liao’s willingness to challenge his peers, referring to the importance of lifestyle to his peers. It is our belief that the outcome of the current discourse on work-life balance will have a profound influence on the nature of the professionalism of the future. Altruism, simply stated as the willingness to consistently put the patient’s interests first, has been a defining characteristic of the medical profession for generations.^8^ A sense of personal entitlement is antithetical to altruism. While older generations can offer opinions and advice, experience has shown that change is largely determined by the generation entering medicine’s community of practice. Obviously some accommodation must be made in response to the demands of the current generation; however, altruism itself must be maintained. Trust is essential to the healing arts and no physician can be deemed worthy of trust if he or she is believed to be putting their personal interests above that of the patient, and if they are shown to lack a commitment to their patients. However, how altruism will be operationalized by current learners is the responsibility of their generation. Thus, Dr. Liao needs to participate in a dialogue with his peers.

Inherent in the letter is also a challenge to the leaders of both undergraduate and postgraduate medicine. Dr. Liao’s letter encourages us to examine our own actions. Are we indeed encouraging students to rationally assess their own attitudes and values in a way that will prepare them for the practice of medicine? Recognizing that we have a very limited ability to alter deeply held generational beliefs, we must ask ourselves whether we are providing a safe space for both intra- and intergenerational discussions on these issues. It is our belief that, as a profession, we have an obligation to do so.
